# Curvature and Temperature Measurement Based on a Few-Mode PCF Formed M-Z-I and an Embedded FBG

**DOI:** 10.3390/s17081725

**Published:** 2017-07-27

**Authors:** Hui Liu, Hangzhou Yang, Xueguang Qiao, Yongqiang Wang, Xiaochong Liu, Yen-Sian Lee, Kok-Sing Lim, Harith Ahmad

**Affiliations:** 1 Physics Department, Northwest University, Xi’an 710069, China; fangzdsz@163.com (H.L.); xgqiao@nwu.edu.cn (X.Q.); yqwang@nwu.edu.cn (Y.W.); 2 National Key Laboratory of Thermostructure Composite Materials, Northwestern Polytechnical University, Xi’an 710072, China; liuchong@nwpu.edu.cn; 3 Photonics Research Centre, University of Malaya, 50603 Kuala Lumpur, Malaysia; yensian1110@gmail.com (Y.-S.L.); kslim@um.edu.my (K.-S.L.); harith@um.edu.my (H.A.)

**Keywords:** Mach-Zehnder interferometers (MZI), few-mode photonic crystal fiber FBG

## Abstract

We have experimentally demonstrated an optical fiber Mach-Zehnder interferometer (MZI) structure formed by a few-mode photonic crystal fiber (PCF) for curvature measurement and inscribed a fiber Bragg grating (FBG) in the PCF for the purpose of simultaneously measuring temperature. The structure consists of a PCF sandwiched between two multi-mode fibers (MMFs). Bending experimental results show that the proposed sensor has a sensitivity of −1.03 nm/m^−1^ at a curvature range from 10 m^−1^ to 22.4 m^−1^, and the curvature sensitivity of the embedded FBG was −0.003 nm/m^−1^. Temperature response experimental results showed that the MZI’s wavelength, λ_a_, has a sensitivity of 60.3 pm/°C, and the FBG’s Bragg wavelength, λ_b_, has sensitivity of 9.2 pm/°C in the temperature range of 8 to 100 °C. As such, it can be used for simultaneous measurement of curvature and temperature over ranges of 10 m^−1^ to 22.4 m^−1^ and 8 °C to 100 °C, respectively. The results show that the embedded FBG can be a good indicator to compensate the varying ambient temperature during a curvature measurement.

## 1. Introduction

Optical fiber sensors have attracted significant research interest and have been widely used to monitor the health of smart engineering structures due to their unique advantages of compact size, low cost, high sensitivity, and immunity to electromagnetic interference [1,2,3,4].Curvature, bending, and strain of engineering structures are important parameters closely associated with the mechanical loading and health condition of the structures. Many sensors based on optical fibers have been proposed for bending or strain measurement, such as Long period Grating (LPG) [5,6], FBG [7,8,9], and Mach-Zehnder interferometers [10,11,12,13], etc. Two LPGs fabricated in hollow eccentric optical fibers under different laser irradiation directions possess the detection ability for the bending direction. However, its sensitivity is lower than that of the LPGs in single-mode fibers [5]. It has been extensively studied [14] and improved in terms of accuracy and resolution [15]. For an optical fiber Mach-Zehnder interferometer (MZI) structure, two coupling elements in the structure will split and recombine the higher-order cladding modes to the core mode in order to form the interference pattern. Shen et al. have demonstrated a curvature sensor based on a polarization-dependent in-fiber MZI with a maximum sensitivity of 0.882 dB/m^−1^ for the curvature measurement in the range of 0.1 m^−1^ to 0.35 m^−1^ [16]. Zhang et al. have proposed a curvature sensor based on an in-line MZI formed by cascading two hump-shaped tapers. It has recognizable features for different bend directions, as well as high sensitivities for ambient temperature and axial force. For a sensor length of 13.4 mm, the sensitivities are 10.224 and −4.973 nm/m^−1^ for opposite bending directions of 0° and 180°, respectively [17]. For a sensor length of 20 mm, the device has a curvature sensitivity as high as −22.227 nm/m^−1^ in the range of 4.66–5.50 m^−1^, and a temperature sensitivity of 0.0847 nm/°C in the range of 60 °C to 100 °C [18].

In recent years, lateral offset splicing of PCF with single-mode fiber (SMF) has been employed to excite higher-order cladding modes for bending measurement [19,20,21]. The core offset technique is used for excitation of the cladding modes and mode coupling between the cladding modes and the core modes. The use of PCFs as sensing elements is generally achieved by transferring a macroscopic deformation into the fiber axial strain, which then translates into wavelength shifts in the output spectrum. An MZI made of a piece of seven cores of PCF sandwiched between two SMFs with lateral offset splicing was made into a directional bending vector sensor. Bending sensitivity of 1.232 nm/m^−1^ has been obtained experimentally [21]. In 2015, Huang et al. reported the effect of bending on a six-air-hole grapefruit PCF. The PCF was spliced to SMF with a lateral offset and a micro-bending sensitivity of −0.754 nm/m^−1^ has been observed [22].

In this paper, we report an MZI-based sensor for curvature measurement. Unlike other curvature sensors, lateral core offset is not used for sensitivity enhancement because of the high attenuation loss to the core mode and the weakened mechanical strength of the fiber. In the fabrication, the MZI structure was formed by sandwiching a few-mode PCF between two MMF segments in which the MMF segments play the role in the excitation of higher-order core modes in the MZI. An FBG was inscribed in the PCF core to compensate for the temperature effect occurring in the measurement. In the characterization of the MZI, the effect of the MZI length on the sensor’s sensitivity was investigated. It was observed that the embedded FBG was not sensitive to the bending. Hence, the Bragg wavelength of the FBG can provide an accurate temperature reading and reduces the cross-effect of temperature in the curvature measurement.

## 2. Design and Theoretical Analysis

**Figure 1:**
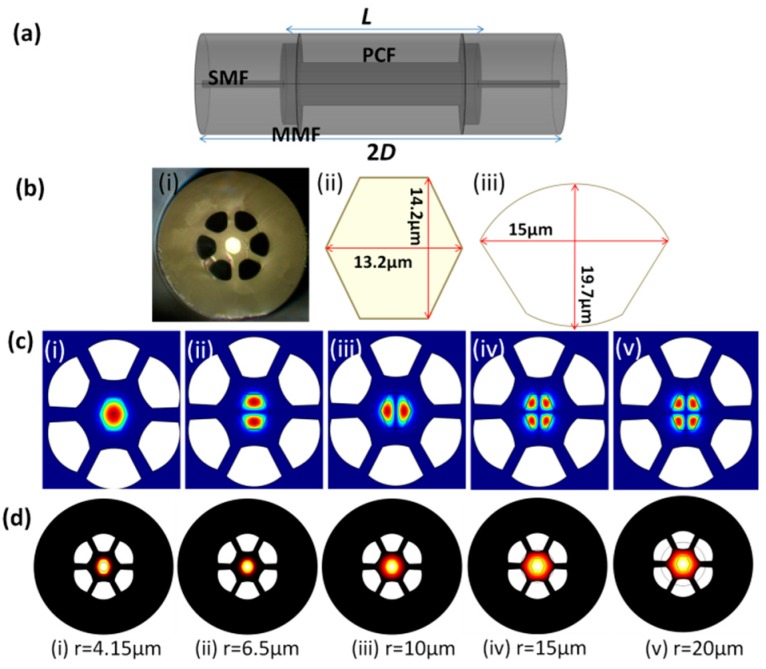
(**a**) Schematic of the proposed MZI structure; (**b**) (i) The optical micrograph of the PCF’s cross section, (ii) The dimension of the core, and (iii) the dimension of the air hole; (**c**) The simulated mode field distributions in the core of PCF at 1550 nm: (i) LP_01_ mode, (ii) and (iii) LP_11_ modes, (iv) and (v) LP_21_ mode; (**d**) The effect of LP_01_ input beam radius, *r*, on the mode profile in the PCF.

In this work, a MZI device is formed by splicing both ends of a few-mode PCF to an MMF as illustrated in Figure 1a. The MZI structure is here with known as MPM. The PCF, denoted as (P), is sandwiched between two MMF segments (M). Each of the MMF segments (core/cladding diameter of 105/125 μm) spliced to the two ends of the PCF has an approximate length of 140 μm. The SMFs with a core/cladding diameter of 8.2/125 μm is spliced to the MMFs to the lead-in and lead-out the light through the MPM. The role of the first MMF segment is to couple and excite higher-order core modes into the PCF. At the second MMF segment, intermodal interference among the higher-order modes happens when they are recombined and coupled into the lead-out SMF. The output spectrum is then guided by the lead-out SMF towards an optical sensing interrogator (OSI) for measurement. In Figure 1b(i) the cross-section of the used PCF is shown, which has a germanium-doped (Ge-doped) core with a refractive index (RI) of 1.473. The diagonal length of the core is 13.2 μm and the height of the core is 14.2 μm as shown in Figure 1b(ii). Several spatial modes can be supported in this core. There are six air holes surrounding the core and each air hole has a dimension of radial and hole-spacing of 15 μm and 19.7 μm, respectively. The cross-section and dimension of the air hole areas shown in Figure 1b(iii). The gap between the adjacent air hole is 7.7 μm, and the outer cladding diameter of the PCF is 125 μm.

The propagation of various higher-order modes in the PCF was simulated by using COMSOL’s (COMSOL Inc., Stockholm, Sweden) finite element method (FEM) and the results are as presented in Figure 1c. The figures show the guided mode field distribution in the few-mode PCF at 1550 nm. From the simulation results in Figure 1c, it can be seen that the few-mode PCF has the ability to support the few core modes in the fiber core due to its large core diameter (large V-number). In addition, we have employed the simulator to investigate the effect of input beam radius on the mode field distribution in the PCF. Fundamental modes with different beam radii were launched into the PCF in the simulation. The input parameters for beam radius were 4.15 μm (size of SMF core), 6.5 μm (size of PCF core), 10 μm, 15 μm, and 20 μm. For each case, the z-component of the power density distribution across the fiber core and cladding has been simulated and they are as shown in Figure 1d. It is observed that the change in the input beam radius influences the mode field distribution over the fiber cross-section. For an input beam radius of 4.1 μm and 6.5 μm, the excited beams are confined within the PCF core and they share the hexagonal shape of the core. As the input beam radius increases, the mode profile extends into the cladding region of the PCF (see Figure 1d (iii)–(v)). This leads to the excitation of higher-order core modes which are more sensitive to the fiber curvature. However, the size of the mode profile is constrained by the air holes surrounding the core when the input beam radius is increased above 20 μm.

The use of MMF in MZI structure is discussed below. In general, intensity of the transmission spectrum of a MZI is given by [23]:

(1) $$I=I_{co}+I_{cl}+2\sqrt{I_{co}I_{cl}}\cos(\Delta \varphi )$$

where *I* is the intensity of the interference, *I_co_* and *I_cl_* are the intensities of the core mode and higher order core mode, respectively, ϕ is the corresponding phase difference between the core mode and the higher-order core mode. We can see that the visibility of the interference *I* is optimum when *I_cl_* = *I_co_*. The increase in the visibility of *I* will enhance the sensor’s detection resolution significantly. However, a large portion of light power is carried by the core mode in an SMF. Direct splicing the SMF with the PCF will lead the excitation of the core mode and low-power distribution to the higher-order modes. Therefore, we employed the first MMF to act as the beam expander so that more light power can be distributed to the higher order core mode in the PCF. The role of the second MMFs is to combine and couple the higher-order core modes from the PCF into the lead-out SMF. This helped improve the visibility of the fringe patterns in the output spectrum.

The distribution of energy and the visibility of fringe pattern are heavily depending on the ratio of the intensity of *I_LP_*_01_ and *I_LPij_*. Thus, the transmitted spectrum intensity, *I*, of the proposed MZI structure can be expressed as [23]:

(2) $$I=I_{01}+\sum_{ij}I_{ij}+2\sum_{ij}\sqrt{I_{01}I_{ij}}\cos(\Delta \varphi_{ij})$$

where *I*_01_ is the intensity of the fundamental mode, *I_ij_* the higher-order core mode, *ϕ_ij_* is the corresponding phase difference between the core mode and the *i*^th^ higher-order core mode. Here *i*, *j* = 1, 2, 3... The optical path length difference between the fundamental mode, *I_LP_*_01_, and a higher-order core mode, *I_LPij_*, propagating through an interferometer of length *L* at operation wavelength, λ*_m_*, which is the wavelength of light in a vacuum, will lead to a phase difference of [23]:

(3) $$\Delta \varphi_{ij}=\frac{2\pi \Delta n_{eff}^{ij}L}{\lambda_{m}}=(2m+1)\pi$$

where *m* is the order of the mode and $\;\Delta n_{eff}^{ij}$ is the difference between the effective refractive indices of the two modes. In such, interference is observed in the transmission spectrum, for which the *m^th^*-order wavelength dip is located at [23]:

(4) $$\lambda_{m}=\frac{2\Delta n_{eff}^{ij}L}{2m+1}$$

When the MPM structure is bent, the effective indices of fiber core mode and higher order core modes are varied due to the stress-optic effect. The strain difference between the higher order core and core due to the curvature is given by [16]:

(5) Δε=l·C

where *l* is the distance between the higher order core and core while *C* is the curvature. The effective index difference is [16]:

(6) $$\Delta n_{eff}^{ij}=n_{eff}^{01}-n_{eff}^{ij}+k\Delta \varepsilon$$

where *k* is the stress-optic coefficient of the optical fiber. From Equation (4) we can calculate the free spectral range (FSR) between two interference minima by using the following approximation [24]:

(7) $$FSR\approx \frac{\lambda_{1}\lambda_{2}}{\Delta n_{eff}^{ij}L}$$

where λ_1_ and λ_2_ are the detected wavelengths. When the MPM structure is bent, a shift is induced in λ_m_ because $\Delta n_{eff}^{ij}$ is proportional to the change in curvature. From Equations (2)–(4), we can see that the characteristics of the interference fringe patterns depend on the length of MPM, *L*. Meanwhile, Equation (7) indicates that the FSR is inversely proportional to both $\Delta n_{eff}^{ij}$ and the length of the MPM. To achieve high sensitivity in the proposed sensor, the length of MPM should be optimized in order to increase the visibility and FSR of the interference pattern.

**Figure 2:**
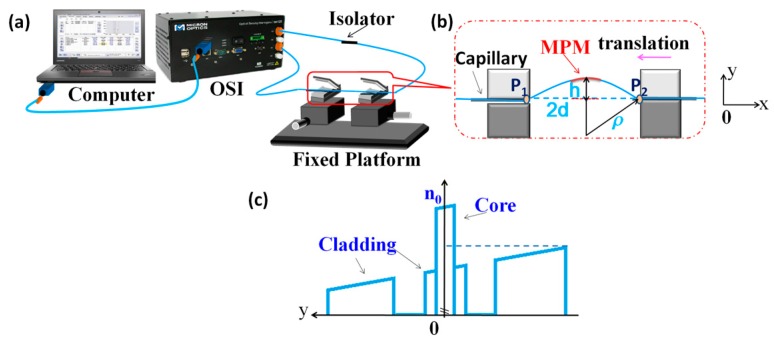
(**a**)Schematic diagram of the experimental setup; (**b**) the configuration used for the bending measurements; and (**c**) RI profile of the curved PCF.

Figure 2a shows the experimental setup for the curvature measurement. The integrated broadband light source of the OSI was used for illuminating the MPM structure in the wavelength range of 1510 to 1590 nm. The input light was first launched into an isolator before it was guided to the MPM to prevent any back reflection to the source. The MPM was positioned on a platform where the orientation and bending diameter of MPM were manipulated. The transmission spectrum of the MPM was analyzed and recorded by the OSI at a wavelength resolution of 1 pm. Figure 2b illustrates the arrangement of the MPM on the platform for bending measurement. Both ends of the MPM structure were inserted through two metallic capillary tubes which were clamped on two translational stages. Both tubes have a length of 2 cm and inner diameter of 300 μm. The fiber was adhered onto the inner side of the tubes at point P_1_ and P_2_ using epoxy to avoid possible twisting of the fiber during the bending experiments. In order to ensure the entire MPM structure subjected to a same curvature, 15 cm long lead-in/lead-out SMF segments were reserved for sides of the MPM. Calibration procedure was implemented before each measurement test for the MPM. The MPM structure was gently straightened by adjusting the position of the translational stages. During the bending calibration process, the ambient temperature was monitored by a thermometer and maintained at 10 ± 1 °C.

The bending of a fiber will affect the fiber’s RI distribution, in which the RI in the compressed section of the fiber will increase, while that in the stretched region will decrease. This encourages the intermodal coupling in the MPM structure and a portion of core modes will be coupled into higher order modes in the PCF core. Figure 2c shows the RI profile of the curved PCF in the MPM structure in Figure 2b. The RI profile is modified from the bent waveguide through a conformal transformation [16]. From the figure, it can be seen that the RI profiles of the core and cladding are tilted due to bending strain that gradually varies across the bending region. The cladding region experiences larger RI change as compared to the core region where the RI of the core region remains almost constant. This is because the fiber cladding suffers more stress as compared to the fiber core. When *y* << ρ, RI profile of curved PCF can be approximated by [25]:

(8) $$n_{y}=n_{0}(1-yC)$$

where *n_y_* is the effective index of the MPM at different positions along the *y*-axis, *n*_0_ is the initial RI along the *y*-axis of the straight PCF, and *C* is the curvature of the PCF. For the bending shown in Figure 2b, RI is decreasing in the +*y*-direction while increasing in –*y*-direction. One of the translational stages is moved closer to another one to induce the bending of the MPM structure in the *y*-direction. In order to determine the bending radius, the bending curve of the fiber is approximated by the arc of a circle. The resulting curvature (*C*) of the MPM structure is estimated by the following equation [25]:

(9) $$C=\frac{1}{\rho }=\frac{2h}{\left(h^{2}+d^{2}\right)}$$

where ρis the curvature radius of the bending, *d* is the half of the distance between P_1_ and P_2_ when the MPM is bent, and *h* is the bending displacement. The half of the distance between P_1_ and P_2_ before the MPM is bent is denoted as *D*. As the fabricated MPM was highly sensitive to bending, the curvature could be expressed in terms of *D* and *d* as in Equation (10) by adopting the approximation of the cos(*D*/ρ) to the fourth-order of the binomial into Equation (9) [25]:

(10) $$\rho^{2}=\rho^{2}\cos^{2}(\frac{D}{\rho })+d^{2}$$

(11) $$C=\frac{1}{\rho }=\frac{\sqrt{3(D^{2}-d^{2})}}{D^{2}}$$

## 3. Fabrication

**Figure 3:**
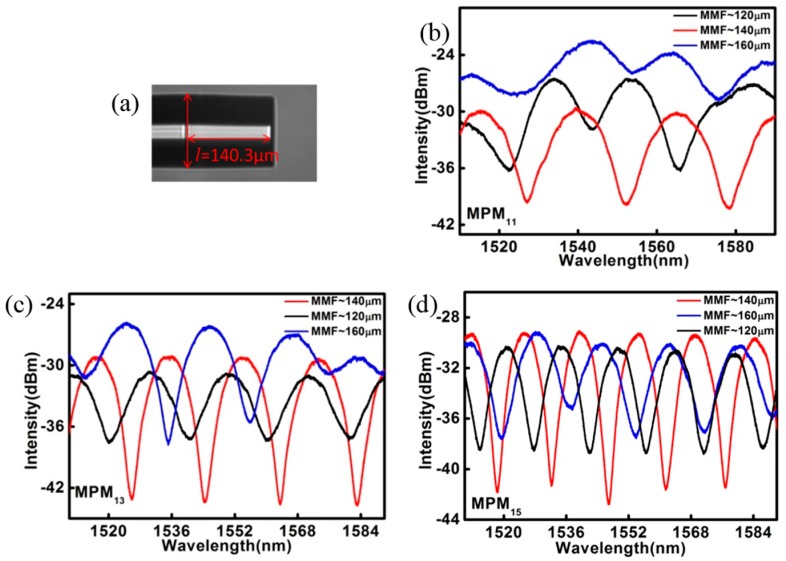
(**a**) The micrograph of MMF, and the transmitted spectra with different lengths of MMF for (**b**) MPM_11_; (**c**) MPM_13_; and (**d**) MPM_15_.

An MPM with various lengths was fabricated by splicing the SMFs, MMFs, and PCF of different segment lengths using an arc fusion splicer (FSM-100P+, Fujikura, Tokyo, Japan). The arcing condition was manually controlled to avoid the collapse of the air holes of the PCF. An optical microscope and the displacement of the machine were utilized to control the length of the MMFs and PCF during the fiber cleaving process. A photograph of the MMFs was captured by the microscope and the length of the MMF was measured from the micrograph. The diameter of the SMF, which is ~125 μm, was used as reference. A typical micrograph of the MMF is shown in Figure 3.

**Figure 4:**
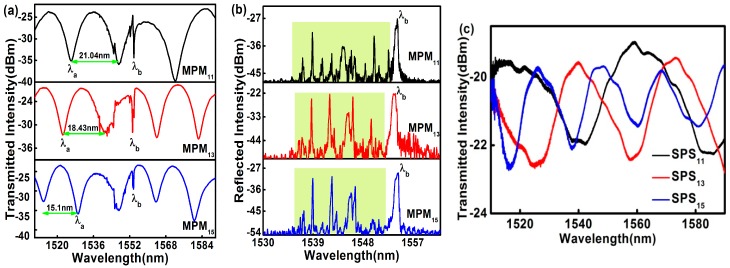
Spectra of MPMs cascaded with a FBG: (**a**) transmission spectra; (**b**) reflection spectra; and (**c**) the transmission spectra of SPS.

The transmission spectrum of the fabricated MPM was then examined and only those that produced satisfying interference fringes in the transmission spectrum were used for the study. Three MPMs with length *L* of 11.3 mm, 13.3 mm, and 15.5 mm were used in this work and they are herewith denoted as MPM_11_, MPM_13_, and MPM_15_, respectively. It should be noted that *L* is the combined length of the PCF and MMF segments. In the experiment, MMFs with different lengths were used to form the MPM structure and the transmission spectra of the MPMs were recorded and shown in Figure 3b–d. The spectra for different MPMs and MMF lengths indicate that MMFs with length of ~140 μm led to the output spectra with optimal visibility. The transmission spectra of MPMs are shown in Figure 4a. It was observed that MPM with PCF’s length in the range of 11 mm to 15 mm produced the most satisfying transmission spectrum and, therefore, they had been chosen for the investigation of their sensing characteristics.

In order to achieve the simultaneous measurement of curvature and temperature, FBG with a length of 10 mm was inscribed in the Ge-doped PCF core of the MPM. Before the grating inscription, the MPMs were hydrogenated for 10 days under a pressure of 13.8 MPa at room temperature. A continuous-wave (CW) frequency-doubled argon ion laser operating at the wavelength of 244 nm was used in conjunction with a phase mask for the FBG writing. Average output power of the laser was ~28 mW and a total UV fluence of ~18.0 J/cm^2^ was applied to produce grating with a transmission dip of ~36 dB. The grating strength decayed to ~21 dB after out-diffusion of residual hydrogen from the PCF. The transmission and reflection spectra of the MPMs with embedded Bragg gratings are shown in Figure 4a,b. It can be seen in Figure 4a that the Bragg wavelength, *λ*_b_, of the FBGs inscribed in the MPMs share the same value and are well embedded in the interference fringe patterns. The same figure also shows that the FSR is decreasing when the length of the MPMs increases from 11.3 mm to 15.5 mm. They are 21.023 nm, 18.43 nm, and 14.97 nm for MPM_11_, MPM_13_, and MPM_15_, respectively. On the other hand, the excited higher-order core modes in the MPM can be easily observed in the reflection spectra as shown in Figure 4b. Based on the prominent peaks observed in the short wavelength region left to *λ*_b_ (green region), it is suggested that the excited higher-order core modes that satisfied the phase-matching condition yield high reflectivity. These core modes in the MPMs are responsible for the small distortion in the transmission spectra in wavelength range near *λ*_b_.

To examine the influence of the MMFs in the MPM structure, a new fiber structure based on the SMF-PCF-SMF (SPS) configuration was made and the reflection spectrum was analyzed. It is observed that the excited higher-order core modes in the SPS were much lower than the MPM structure and the SPS structure has interference fringe patterns with lower visibility. The transmitted spectra of SPS structures are shown in Figure 4c. Evidently, MMFs play an important role in exciting the core modes in the core of the PCF.

## 4. Results and Discussion

**Figure 5:**
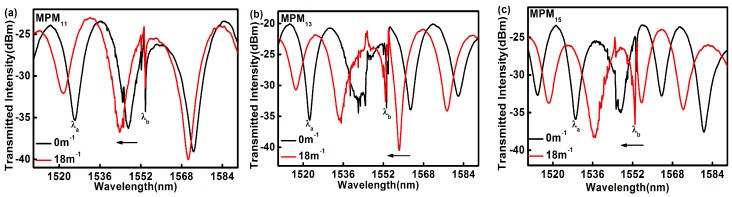
The spectra shift for curvatures of 0 m^−1^ and 18 m^−1^: (**a**) MPM_11_; (**b**) MPM_13_; and (**c**) MPM_15_.

To determine the curvature responses of MPM_11_, MPM_13_, and MPM_15_, the bending curvature of the MPMs was gradually increased and the transmission spectra were recorded at each increment. In the experimental process, the fiber was controlled by the translation platform for the bending measurement. At each bending curvature, the bending condition was held for 10 min to achieve stability before the reading was recorded. The transmission spectra of the MPMs at two different curvatures are shown in Figure 5, where the Bragg wavelength is marked as *λ*_b_ and the MZI’s resonance wavelength is marked as *λ*_a_. It can be observed that the change in *λ*_b_ is very small when the curvature of the MPMs changed from 0 m^−1^ to 18 m^−1^. This might be due to the fact that the RI of the fiber core (where the FBG is inscribed) is not significantly affected by the bending of the fiber, as shown in Figure 5c. As such, the results in Figure 5 strongly suggest that the proposed sensor can perform a reliable curvature measurement in a temperature-varying environment.

**Figure 6:**
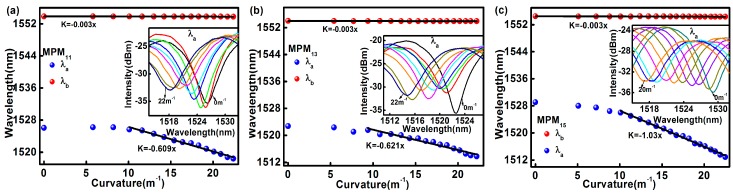
The curvatures characterization of *λ*_a_ and *λ*_b_: (**a**) MPM_11_; (**b**) MPM_13_; and (**c**) MPM_15_.

*λ*_a_ and *λ*_b_ obtained from the transmission spectra of MPM_11_, MPM_13_, and MPM_15_ at different curvatures are shown in Figure 6a–c, respectively. The inset in these figures shows the shift in *λ*_a_ in the transmission spectra when different curvatures were applied. The results show that the MZI spectra are very sensitive to curvature and the spectra are blue-shifted as the curvature increases. The change of *λ*_b_ with respect to the curvature is shown as red dots in Figure 6. *λ*_b_ has very low sensitivity of −0.003 nm/m^−1^ towards the curvature change. On the other hand, blue markers in Figure 6 are the MZI’s responses to different applied curvatures. It is found that *λ*_a_ (blue markers) is linearly proportional to the MPM curvature ranges from 10 m^−1^ to 22.4 m^−1^. From the linear fitting equations, it can be seen that the proposed sensor has a curvature sensitivity of −0.609 nm/m^−1^, −0.621 nm/m^−1^, and −1.03 nm/m^−1^ for MPM_11_, MPM_13_, and MPM_15_, respectively. This shows that the curvature sensitivity increases when the MPM length increases from 11 mm to 15 mm, where the curvature sensitivity of MPM_15_ is 341 times higher than that of the FBG.

**Figure 7:**
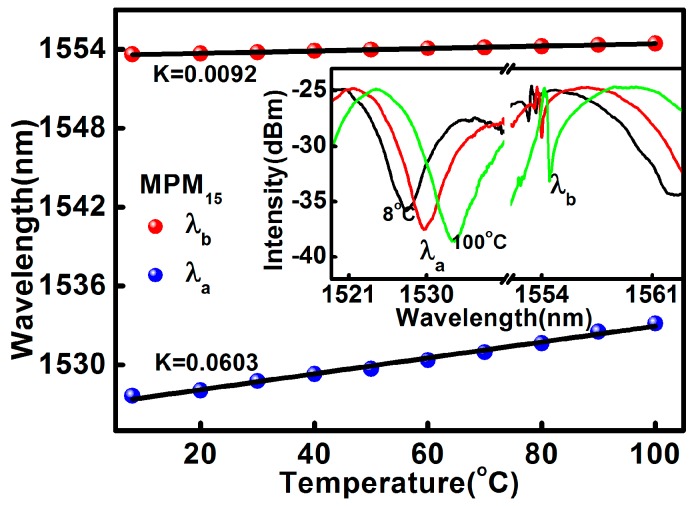
The temperature characterization of MPM_15_.

The temperature response of the proposed sensor at the MPM curvature of 10 m^−1^ has also been investigated. The proposed sensor was fixed at a curvature of 10 m^−1^ and the fixed platforms together with the proposed sensor were placed in an oven to test the temperature response. The temperature of the oven was increased from 8 °C to 100 °C where the increment was programmed to be 10 °C per step. The temperature of the oven was kept at each step for 15 min in order to obtain a homogeneous temperature distribution across the fiber in the oven. During the heating process, the temperature of the oven and the transmission spectra of the sensor were recorded. The effect of temperature on the spectra is presented in Figure 7. Both *λ*_a_ and *λ*_b_ have linear temperature responses in the range of 8 °C to 100 °C. However, *λ*_a_ has better temperature sensitivity than that of the FBG, *λ*_b_. The measured temperature sensitivities for *λ*_a_ and *λ*_b_ are given by 60.3 pm/°C and 9.2 pm/°C, respectively.

From the aforementioned results, it is clear that the proposed sensor can be used for simultaneous curvature and temperature measurement. Generally, when the curvature and temperature are simultaneously applied to the sensing head, the relationship between the wavelength shift, ∆λ*_i_* and the variations of curvature and temperature, ∆*C* and ∆*T*, can be expressed as [24]:

(12) $$\Delta \lambda_{i}=K_{Ti}\Delta T+K_{Ci}\Delta C$$

where *i* = {1,2} in which 1 corresponds to the resonance wavelength of MPM, *λ_a_* and 2 corresponds to the Bragg wavelength, *λ_b_* meanwhile K_T*i*_ and K_C*i*_ are the corresponding temperature and curvature sensitivities, respectively, where *K_T_*_1_ = 0.0603 nm/°C, *K_C_*_1_ = −1.03 nm/m^−1^, *K_T_*_2_ = 0.0092 nm/°C, and *K_C_*_2_ = −0.003 nm/m^−1^. Finally, the characteristic matrix equation for simultaneous measurements of temperature in the range of 8 °C to 100 °C and curvature in the range of 10 m^−1^ to 22.4 m^−1^ can be obtained from Equation (12) as follows:

(13) $$\left[\begin{array}{c} \Delta T\\ \Delta C \end{array}\right]=\left[\begin{array}{cc} -0.32 & 110.81\\ -0.99 & 6.49 \end{array}\right]\left[\begin{array}{c} \Delta \lambda_{1}\\ \Delta \lambda_{2} \end{array}\right]$$

## 5. Conclusions

We have reported a bending measurement device based on an MZI structure. The structure was produced by splicing a piece of PCF with MMFs, of which the SMFs were spliced to both ends of the MZI structure to lead-in and lead-out the guided light. The incorporation of two 140-μm long MMF segments in the fiber structure produces an output spectrum with improved interference visibility which enhances the measurement accuracy. An FBG was inscribed in the photosensitive core of PCF for temperature compensation. Experimental results show that the curvature sensitivity increased with the length of MZI. Among the three MPMs that were investigated, MPM_15_ yields the best sensitivity of −1.023 nm/m^−1^ in curvature range from 10 m^−1^ to 22.4 m^−1^. The embedded FBG has curvature sensitivity of 0.003 nm/m^−1^, which is much lower than that of the MZI during curvature measurement. Temperature test results shows that the proposed structure has temperature sensitivities of 60.3 pm/°C and 9.2 pm/°C for MZI and FBG, respectively, over the temperature range of 8 °C to 100 °C. It is shown that the MZI’s wavelength shift induced by the change of temperature during the curvature measurement can be precisely measured by the FBG to achieve a simultaneous measurement of the curvature and the temperature based on a 2 × 2 characteristic matrix. It is a small and compact device suitable for curvature measurement or characterization testing for any mechanical structures.
